# Exposure to hand-held mobile phone use while driving among Iranian passenger car drivers: an observational study

**DOI:** 10.5249/jivr.v4i2.130

**Published:** 2012-07

**Authors:** Ahad Ashrafi Asgarabad, Ahmad Naghibzadeh Tahami, Narges Khanjani

**Affiliations:** ^a^Postgraduate student of Epidemiology, School of Public Health, Kerman University of Medical Science, Kerman, Iran.; ^*b*^Assistant Professor of Epidemiology, School of Public Health, Kerman University of Medical Science, Haft Bagh Alavi Highway, Kerman, Iran.

The use of hand-held mobile phones/cellular phones while driving is a known risk factor for road traffic accidents.^1^ In Iran, the use of mobile phones has increased dramatically in recent years. However, little research has been done to determine the prevalence of mobile phone use while driving in Iran. We conducted an observational, cross-sectional study to document patterns of mobile phone use among drivers of passenger vehicles, including cars, mini-vans, and utility vehicles in the streets of Kerman, a city in the south-east of Iran, in the fall of 2009. Direct observational study is a less biased method of assessing exposure to cellular phone use among passenger car drivers in comparison to self-report data and police accident reports.^2^

The results showed that of the approximately 30,000 drivers who were observed on the weekdays during daytime hours and on different streets, an average of 3.6% were using their hand-held mobile phones while driving. There was no statistically significant difference between men and women in mobile phone use during driving. However, younger (estimated age <30 years) and middle aged drivers (estimated age 30-50 years) used mobile phones more frequently than the older adults and the elderly. Also, the use of mobile phones were more frequent on streets in the central part of town, and the ring road in comparison to suburban streets, and were more frequently used in the morning than in the afternoon.

To our knowledge, this is the first study on this topic that has been conducted in Iran or other countries in the region. However, similar observational findings from Melbourne and Perth in Australia show that between 1.5 to 2% of motor vehicle drivers used a mobile phone while driving.^3,4,5^ Other similar observational studies from Michigan^6^ and Minnesota^2^ have reported that 5.8% and 4.2%-4.7% of motor vehicle drivers, respectively, used hand-held cellular phones while driving.

Additional national level studies are needed to better understand the rate of exposure to hand held cellular phones among drivers of passenger cars in Iran. Our findings show that conversing on a hand-held cellular phone among drivers in Kerman is nearly as popular as it is among their counterparts in Western countries. A culturally tailored tool is needed to assess knowledge and attitudes of Iranian passenger car drivers in respect to the safety concerns of using cellular phones behind the wheel. Results of such studies can offer valuable educational and motivational suggestions for improving prevention and the reduction of cellular phone use among drivers of passenger cars in Iran.
